# The Predictive Role of Prostate-Specific Antigen Changes Following Transurethral Resection of the Prostate for Patients with Localized Prostate Cancer

**DOI:** 10.3390/cancers13010074

**Published:** 2020-12-29

**Authors:** Chun-Te Wu, Yun-Ching Huang, Wen-Cheng Chen, Miao-Fen Chen

**Affiliations:** 1 Department of Urology, Chang Gung Memorial Hospital, Keelung 204, Taiwan; chuntewu@cgmh.org.tw; 2 College of Medicine, Chang Gung University, Taoyuan 131, Taiwan; dr5326@cgmh.org.tw (Y.-C.H.); danielchen@cgmh.org.tw (W.-C.C.); 3 Department of Urology, Chang Gung Memorial Hospital, Chiayi 613, Taiwan; 4 Department of Radiation Oncology, Chang Gung Memorial Hospital, Chiayi 613, Taiwan

**Keywords:** prostate cancer, local treatment, TURP, PSA, conservative management

## Abstract

**Simple Summary:**

A part of localized prostate cancer (PC) was an incidental finding in patients who received transurethral resection of the prostate (TURP) for urinary symptoms. The present study examined whether changes in prostate-specific antigen (PSA) levels after TURP possess a predictive value for localized PC. Our data revealed that patients at intermediate risk who are associated with tumor involvement ≤5% in TURP specimens, PSA_TURP ≤ 4 ng/mL, and ≥68% PSA reduction following TURP might be suitable for conservation management instead of immediate local therapy. Moreover, for patients with no pre-TURP PSA, Gleason score (GS) < 7, and low PSA_TURP could potentially be utilized to select which patients could be considered for conservative management after TURP. The findings suggest the pathologic finding of TURP and changes in PSA could be used as adjuvant markers to guide a risk-adaptive strategy for patients with localized PC.

**Abstract:**

Regarding localized prostate cancer (PC), questions remain regarding which patients are appropriate candidates for conservative management. Some localized PC was an incidental finding in patients who received transurethral resection of the prostate (TURP) for urinary symptoms. It is known that TURP usually affects the level of prostate-specific antigen (PSA). In the present study, we examined whether changes in PSA levels after TURP possess a predictive value for localized PC. We retrospectively reviewed the clinical data of 846 early-stage PC patients who underwent TURP for urinary symptoms upon diagnosis at our hospital. Of 846 patients, 687 had tumor involvement in TURP specimens, and 362 had post-TURP PSA assessment. Our data revealed that, in addition to low GS and PSA levels at diagnosis, ≤5% tumor involvement in TURP specimens, greater PSA reduction (≥68%) following TURP, and post-TURP PSA ≤ 4 were significantly associated with better progression-free survival (PFS). Survival analysis revealed that the addition of prostate-directed local therapy significantly improved PFS in intermediate- and high-risk groups, but not in the low-risk group. Moreover, in the intermediate-risk group, local therapy improved PFS only for patients who were associated with post-TURP PSA > 4 ng/mL or <68% PSA reduction following TURP. We also found that local therapy had no obvious improvement in PFS for those with post-TURP ≤ 4 ng/mL regardless of pre-TURP PSA. In conclusion, conservative management is considered for patients at low or intermediate risk who have greater PSA reduction following TURP and low post-TURP PSA. Therefore, the levels of PSA following TURP might be helpful for risk stratification and the selection of patients for conservative management.

## 1. Introduction

Screening with prostate-specific antigen (PSA) testing has led to the identification of more patients with localized prostate cancer (PC) [1]. For these patients, the risk of failure varies, and management approaches range from active surveillance [2,3] to curative-intent therapy, including radical prostatectomy or definitive radiotherapy [4,5]. Although curative treatment may reduce the risk of cancer progression for some patients, locally aggressive treatment of the prostate is associated with impaired genitourinary function and reduced quality of life. Given the morbidity associated with treatment of PC, active surveillance has emerged as an option for low-risk PC, supported by substantial evidence regarding the favorable outcomes of conservative management [2,6,7]. Although conservative management can reduce overtreatment, disease progression is an issue for localized-PC patients with conservative management [3,5,8]. Notably, it remains unclear which patients are appropriate candidates for conservative management.

In addition to the Gleason score (GS), PSA is used for determining tumor involvement and prognosis [9,10]. The level of PSA is commonly used as a criterion for patients undergoing conservative management [2,11]. The presence of benign prostatic hyperplasia (BPH) or bladder outlet obstruction may lead to moderate elevations in the PSA level [12,13,14]. The PSA level is also related to prostate volume; the decrease in PSA level is proportional to the amount of tissue removed [14,15]. Transurethral resection of the prostate (TURP) is the surgical treatment for symptomatic BPH [16]. A part of localized PC was an incidental finding in patients who received TURP for urinary symptoms. It is known that serum PSA levels usually decrease following a TURP procedure. Therefore, this study investigates whether tumor involvement in TURP specimens, PSA changes following TURP, and post-TURP PSA levels (PSA_TURP) could be used as adjuvant markers to guide a risk-adaptive strategy for localized PC, especially for those who are unable to tolerate the toxicity of curative therapy.

## 2. Results

### 2.1. Predictive Value of Tumor Involvement in Transurethral Resection of the Prostate (TURP)

**Figure 1:**
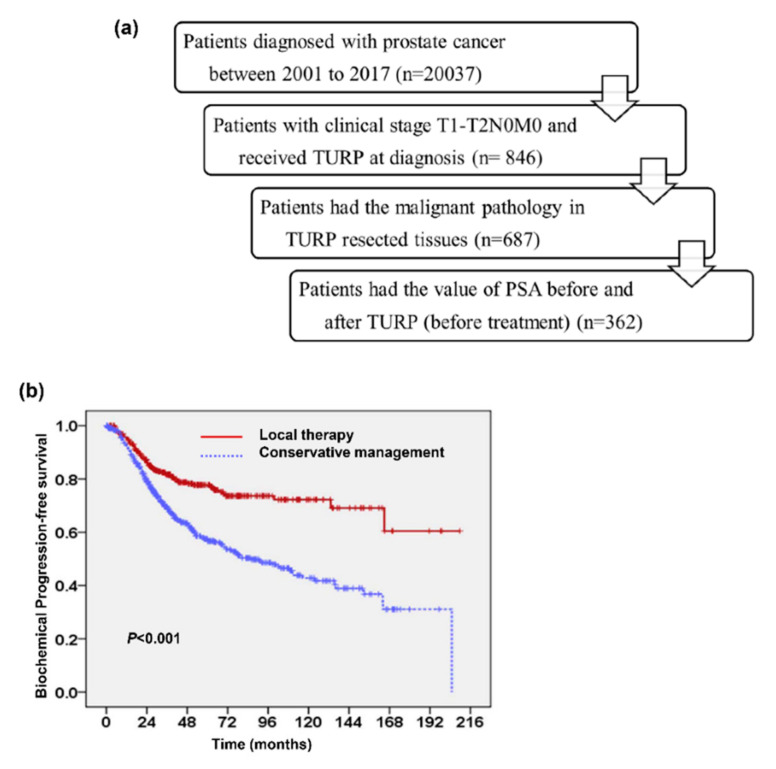
Survival in patients with early stage prostate cancer. We enrolled the patients with clinical stage T1–T2N0M0 prostate cancer who underwent transurethral resection of the prostate (TURP) into our present study. The study flow chart was presented in (**a**). Additional, Kaplan–Meier survival curves of progression-free survival (PFS) stratified by the addition of local therapy (**b**).

**Figure 2:**
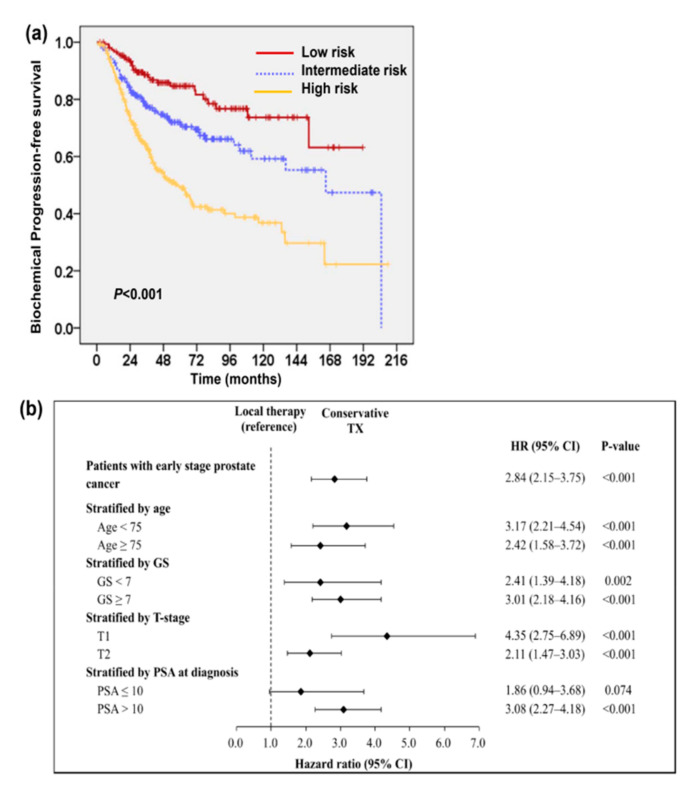
Factors correlated with clinical outcome of patients with localized prostate cancer. Kaplan-Meier PFS survival curves of 687 patients stratified by risk groups (**a**). Additional, local therapy significantly improved PFS when patients were stratified by clinical risk factors (**b**).

**Figure 3:**
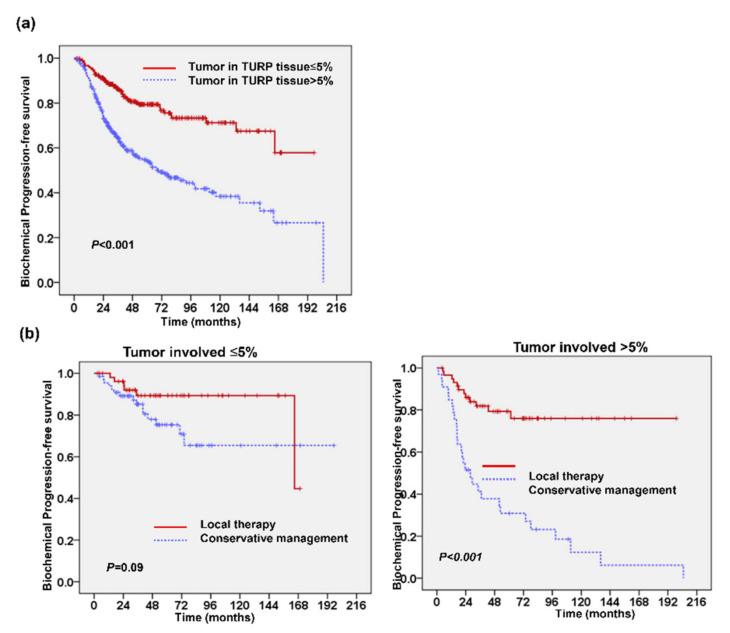
Tumor involvement in TURP prostatic tissue correlated with clinical outcome of patients with localized prostate cancer. Kaplan-Meier PFS survival curves of patients stratified by the percentage of tumor involvement in TURP specimens (**a**). Additional, local therapy significantly improved PFS in patients who produced TURP specimens with >5% tumor involvement but did not significantly benefit those with ≤5% tumor involvement (**b**).

The survival analysis for 846 early-stage PC patients (Figure 1a) showed that the five-year cancer-specific survival (CSS) and progression-free survival (PFS) rates were 92% and 66%, respectively; the 10-year CSS and PFS rates were 82% and 54%, respectively. Univariate analysis showed that GS < 7, PSA at diagnosis (PSA_Dx) ≤ 10 ng/mL, and the addition of local therapy were significant predictors for PFS. Patients who underwent local therapy had significantly improved five-year CSS (96% versus 90%, *p* < 0.001) and five-year PFS (Figure 1b). These patients were further divided into low-risk (GS < 7 and PSA ≤ 10 ng/mL), intermediate-risk (GS ≥ 7 and PSA ≤ 10 ng/mL, or GS < 7 and PSA > 10 ng/mL), and high-risk groups (GS ≥ 7 and PSA > 10 ng/mL). Patients in the high-risk group exhibited poorer PFS than those in the intermediate- or low-risk groups in total or subgroup (local therapy and conservative management) analyses (Figure 2a and Figure S1). Further multivariable analysis based on different covariables showed that the addition of local therapy could reduce the risk of biochemical failure by more than 65% in patients with T1–T2N0M0 PC, and significantly improved PFS relative to conservative treatment when patients were stratified by clinical risk factors at diagnosis (Figure 2b). There were 687 patients appearing with tumor involvement in TURP specimens. We further examined the predictive role of tumor involvement in TURP prostatic tissue. Figure 3a and Table 1 revealed that ≤5% tumor involvement in TURP specimens was significantly associated with a lower biochemical PFS. Moreover, for patients in the intermediate-risk group, local therapy significantly improved PFS in patients who produced TURP specimens with >5% tumor involvement but did not significantly benefit those with ≤5% tumor involvement (Figure 3b).

### 2.2. Changes in Prostate-Specific Antigen (PSA) Following TURP

**Figure 4:**
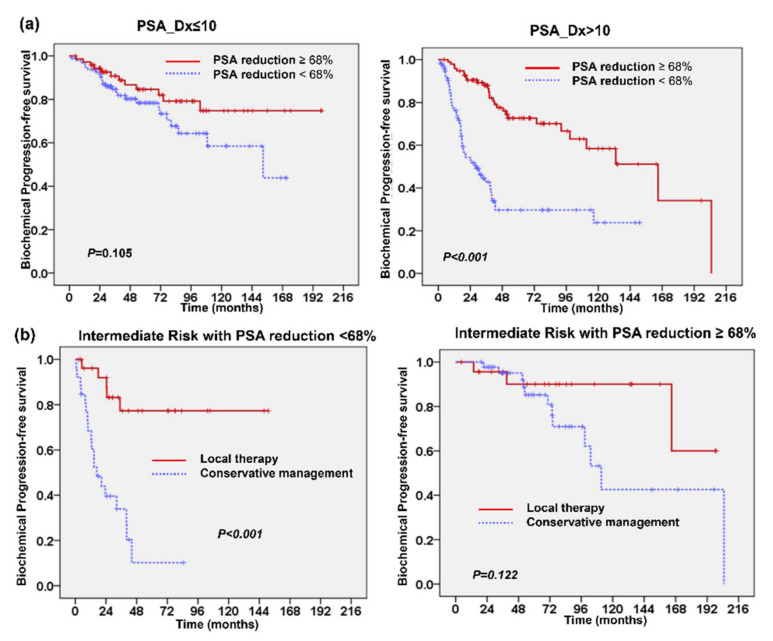
PSA reduction following TURP correlated with clinical outcome of patients with localized prostate cancer. Kaplan-Meier PFS survival curves showed that ≥68% PSA reduction following TURP was associated with better PFS in patients with higher PSA_Dx, but not for patients with lower PSA_Dx (**a**). Additionally, for patients in the intermediate-risk group, local therapy significantly improved the prognosis for patients with the PSA reduction <68%, but not for patients with greater PSA reduction (**b**).

In total, data regarding PSA_Dx and rechecked PSA levels after TURP (PSA_TURP) were obtained from 362 patients. Among these patients, 334 had lower PSA_TURP than PSA_Dx, with a median 66% reduction in PSA level following TURP. To assess whether the change in PSA following TURP had an impact on the prognosis, the level of PSA reduction was redefined as a binary variable by finding the value from a receiver-operating characteristic (ROC) curve that maximized the percentage correctly classified for biochemical failure rate. Accordingly, we divided the patients into two groups with 68% PSA reduction following TURP (67% sensitivity and 62% specificity). In a univariate analysis, patients with <68% PSA reduction had higher rates of biochemical failure rate (78/193 versus 38/169; *p* < 0.001), higher rates of disease failure (38/193 versus 18/169; *p* = 0.018), and poor PFS compared to those with ≥68% PSA reduction (*p* < 0.001). Furthermore, as shown in Figure 4a, ≥68% PSA reduction following TURP was a prognostic predictor for PFS in patients with higher PSA_Dx, but not for patients with lower PSA_Dx. We further examined whether PSA reduction could be utilized to assist with treatment decision for patients in the intermediate-risk group. As shown in Figure 4b, local therapy significantly improved the prognosis for patients with the PSA reduction <68% (*p* < 0.001), but did not significantly affect patients with greater PSA reduction (*p* = 0.122). For high-risk patients, the survival benefit brought about by the addition of local therapy was independent of the PSA reduction level. Based on our data, for patients with early-stage PC at intermediate risk, the level of PSA reduction following TURP could be utilized to assist with patient selection for local therapy.

### 2.3. Predictive Role of PSA Level after TURP

**Figure 5:**
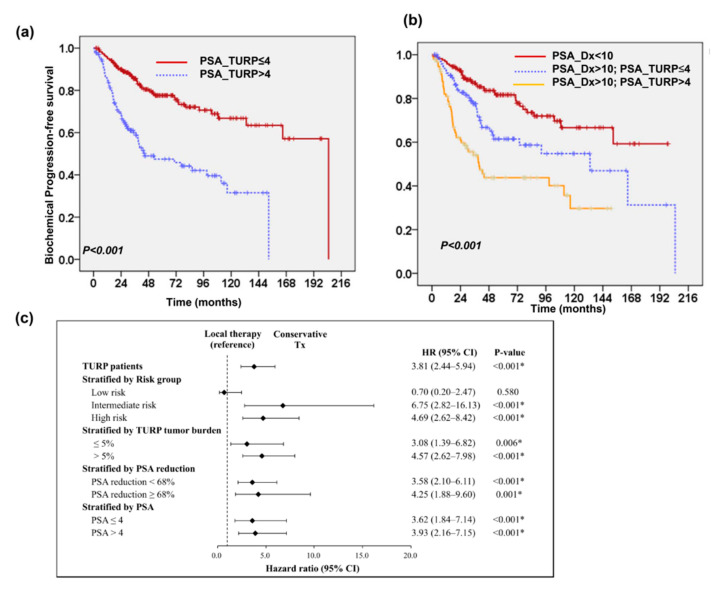
Predictive role of PSA level after TURP. Kaplan-Meier PFS survival curves of 362 patients stratified by PSA levels after TURP ≤ 4 ng/mL (versus > 4 ng/mL) (**a**). Survival difference in patients according to PSA levels at diagnosis combined with that after TURP (**b**). Additionally, local therapy significantly improved PFS when patients were stratified by the pathologic finding of TURP and changes in PSA (**c**). *, Statistical significance.

**Figure 6:**
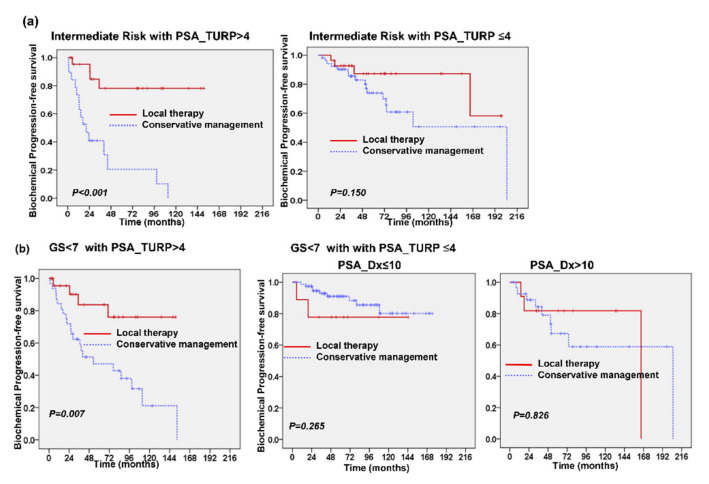
Predictive role of PSA level after TURP in low- and intermediate-risk group. For patients in the intermediate-risk group, local therapy significantly improved the prognosis for patients with high PSA_TURP but not for patients with PSA_TURP ≤ 4 ng/mL (**a**). For patients with GS < 7, local therapy significantly improved the prognosis for patients with high PSA_TURP but not for patients with PSA_TURP ≤ 4 ng/mL regardless of pre-TURP PSA ≤ 10 or >10 ng/mL (**b**).

Among these 362 patients, the median PSA_TURP was 3.77 ng/mL. We further divided into the patients based on PSA_TURP values: high (PSA_TURP > 4 ng/mL) and low (PSA_TURP ≤ 4 ng/mL). The clinical characteristics of both groups are shown in Table 2. The patients in the high PSA_TURP group had higher rates of disease and biochemical failure, as well as poor survival, although more of these patients underwent local therapy (Table 2 and Figure 5a). Furthermore, for these patients with PSA_Dx > 10 ng/mL, survival analysis revealed that patients with lower PSA_TURP had better PFS than patients with higher PSA_TURP (Figure 5b). A multivariate analysis (Figure 5c) showed that the addition of local therapy improved PFS when these patients were stratified by risk group, PSA_TURP, and the PSA reduction following TURP. We also examined the predictive role of PSA_TURP in patients in the intermediate-risk group. We found that local therapy significantly improved the prognosis for intermediate-risk patients associated with high PSA_TURP (Figure 6a). To determine the role of PSA_TURP in assisting with patient selection for avoiding local treatment for patients with low to intermediate risk, we examined the survival benefit brought about by local therapy in 185 patients with GS < 7. A survival analysis revealed that local therapy improved PFS for patients with PSA_TURP > 4 ng/mL, but led to no obvious improvement in PFS for patients with PSA_TURP ≤ 4 ng/mL regardless of pre-TURP PSA ≤ 10 or > 10 ng/mL (Figure 6b). Accordingly, we suggest that PSA_TURP can assist with patient selection, ruling out local treatment for some patients with incidental finding of localized PC and who had no pre-TURP PSA.

## 3. Discussion

Traditionally, clinical and pathologic staging systems were used alone to categorize outcomes, but now cancer is often evaluated using risk-stratification systems. The appropriate treatment policy for localized PC requires further investigation. Local treatment of PC is associated with a reduced quality of life, so these risks must be carefully balanced against any benefit in terms of survival [6,17]. The published literature has shown no significant differences in survival outcomes for patients with early-stage disease treated with various types of local treatment. There is evidence of the benefits of local therapy for intermediate-risk localized PC rather than low-risk PC [2,7,18]. Conservative management is considered a safe alternative to immediate local therapy for selected patients to avoid the potential morbidities associated with available therapies. PC may occur as part of the aging process in men; thus, the likelihood of PC increases with age, such that it affects approximately 80% of men who are 80–89 years of age [7,19]. Although conservative management can reduce the overtreatment of indolent disease, there is concern regarding an increased risk of metastatic disease [3,5,8]. During conservative management, changes in PSA levels are used as markers to predict disease progression and determine whether interventions are appropriate [15,20]. In our study, the overall 10-year CSS was 82%. The addition of local therapy significantly improved CSS and PFS relative to conservative management. Further analysis based on different covariables showed that GS < 7, pretreatment PSA ≤ 10 ng/mL, and the addition of local therapy were associated with better PFS. There is evidence that it is safe to use conservative management with patients with low-risk PC. The most commonly used definition for low risk is PSA ≤ 10 ng/mL, disease stage T1–T2, and GS ≤ 6. Our data showed that local therapy significantly improved PFS in the intermediate- and high-risk groups, but not in the low-risk group. Therefore, for patients with PC in the low-risk group, conservative management is considered instead of local therapy.

The PSA level can increase due to many causes [21]. The PSA level is directly linked with bladder outlet obstruction, prostate volume, and BPH-related events [22]. There is a high prevalence of prostatic hyperplasia in older men, and BPH could induce the elevation of PSA levels as well as PC [15,23]. Therefore, PC patients who have prostatic hyperplasia typically exhibit higher PSA levels at diagnosis; these patients have a greater probability of eventually being in the high-risk group. For the elevated PSA induced by BPH, TURP has been reported to normalize dramatically and maintain the PSA level for an extended period of time. In addition, TURP could decrease the tumor burden for patients who had cancers in the transition zone or anterior portion of the prostate. Tumor burden is an important predictor of long-term outcomes for PC patients [24,25]. Previous studies revealed that PSA levels before and after TURP could be used to estimate the residual tumor burden [26,27]. Under the hypothesis that reduction of prostate tissue presented hyperplasia, inflammation, or abnormal cell could influence the extent of PSA decrement, we investigated whether the pathologic finding of TURP and the changes in PSA levels following TURP could be utilized as a risk-adaptive marker for localized PC, and whether some patients could then be potentially eligible for conservative management. In our study, 687 patients underwent TURP before treatment and produced TURP specimens with tumor involvement. A tumor involvement of ≤5% in TURP specimens was significantly associated with lower biochemical failure rate and longer PFS, compared to a tumor involvement of >5% in TURP specimens. It has been reported that PSA_TURP is an independent predictor of residual cancer, and definitive therapy should be considered for patients with stable or elevating PSA following TURP [28,29]. Therefore, reductions in PSA_TURP may be associated with a lower risk for progression, and PSA_TURP may serve as an adjuvant marker to guide subsequent treatment. In our study, among patients who underwent TURP, PSA levels decreased following TURP in 92% of the patients. Furthermore, greater PSA reduction (≥68%) following TURP and PSA_TURP ≤ 4 ng/mL was associated with lower rates of disease failure and biochemical failure, as well as longer PFS. We examined whether the level of PSA reduction and PSA_TURP could guide the implementation of a risk-adaptive strategy for selected PC patients. Based on our findings, local therapy is recommended for patients in the high-risk group. For those in the intermediate-risk group, patients with high PSA_TURP and/or less PSA reduction (<68%) following TURP obtained more benefit from local therapy. A part of localized PC was an incidental finding of TURP. Furthermore, although TURP is not a routine procedure for PC, it may be performed prior to treatment due to urinary symptoms. Therefore, some patients with localized PC might have no pre-TURP PSA.

The limitations of our study are inherent to investigations based on hospital registries. We were unable to ascertain the reasons for the choice of local therapy or conservative management. Furthermore, we could not adjust for potential unmeasured selection biases regarding performance status, access to healthcare, or other patient-related factors. Accordingly, the study was designed to assess changes in PSA after TURP and how they affect selection of patients for conservative treatment, but not to investigate whether local therapy is better than conservative management for patients with early stage prostate cancer.

Our data revealed that local therapy improved PFS for patients with PSA_TURP > 4 ng/mL, but led to no obvious improvement in PFS for patients with PSA_TURP ≤ 4 ng/mL regardless of pre-TURP PSA ≤ 10 or >10 ng/mL Based on our data, PSA_TURP might help rule out local treatment for patients with no pre-TURP PSA.

## 4. Materials and Methods

### 4.1. Study Population and Study Design

Data were obtained from our hospital’s system, a high-quality cancer registry that provides information regarding each patient’s demographics, disease stage, tumor histology, and primary treatment details. This retrospective study was approved by the institutional review board of Chang Gung Memorial Hospital (No. 201800593B0), and a waiver of informed consent was obtained. This study adhered to strict confidentiality guidelines, in accordance with regulations regarding personal electronic data protection. From 2001 to 2017, there were 20,037 patients newly diagnosed with PC at our hospital. We excluded patients with other cancer diagnoses before PC diagnosis and those who had clinical stage T3–T4, with lymph node involvement, or with distant metastasis at diagnosis, and those who received chemotherapy or hormone therapy prior to reaching biochemical failure. We included 846 patients who were diagnosed with clinical stages T1–T2N0M0 and underwent TURP prior to definite treatment for urinary symptoms; they then underwent follow-up at our hospital. The timing of these enrolled patients receiving TURP was simultaneous with prostate biopsy (85%), and within two months (15%). As shown in Figure 1a, 846 patients with a histologically confirmed diagnosis of prostate adenocarcinoma at clinical stages T1–T2N0M0 were enrolled in our study; all underwent TURP at diagnosis. Patient disease and treatment characteristics are shown in Table 3. At our institution, men were assessed via prostate biopsy if they had PSA levels > 4 ng/mL. Of the 846 patients who underwent TURP, 191 (22.5%) had acute urinary retention (postvoiding urine volume ≥100 mL), 687 produced TURP specimens with tumor involvement (Table S1), and 362 underwent follow-up PSA assessment at 1~2 month after TURP [22,30]. The Gleason sum (GS) was determined by the pathologists based on the 1993 World Health Organization (WHO) consensus conference and 2004 WHO Classification of Tumours of the Urinary System and Male Genital Organs. When a discrepancy in GS occurred between biopsy and TURP specimens, the higher GS was chosen. To assess the role of local therapy in the prognosis of early-stage PC, enrolled patients were divided into two groups based on the management after diagnosis: the local therapy group and the conservative management group. The local therapy group included patients who underwent prostatectomy or definitive radiotherapy as initial treatment after diagnosis; and the conservative management group included patients who did not immediately undergo local therapy after diagnosis and underwent follow-up at our hospital. The main end points were biochemical failure, prostate cancer-specific survival (CSS) (time elapsed between diagnosis and death from PC), and biochemical progression-free survival (PFS) (time elapsed between diagnosis and biochemical failure or death from any cause). Biochemical failure was defined as PSA > nadir + 2 ng/mL (radiotherapy), >0.2 ng/mL (prostatectomy), or the start of salvage treatment for the local therapy group. For the conservative management group, the biochemical failure events were defined as PSA elevation rate > 2 ng/mL per year, or the start of treatment (including local therapy, hormone therapy, or chemotherapy). Furthermore, disease failure was defined as documented loco-regional recurrence and/or distant metastases.

### 4.2. Statistical Analysis

We used the Kaplan‒Meier method for survival curves, and the log-rank test to determine differences in survival curves between groups. Cox proportional hazards models were used for hazard ratios with 95% confidence intervals (CIs) after adjustment for clinical characteristics. All analyses were conducted using SAS statistical software, version 9.2 (SAS Institute, Cary, NC, USA), and SPSS version 17.2 for Windows (SPSS Inc., Chicago, IL, USA).

## 5. Conclusions

The findings indicate the predictive value of the pathologic finding of TURP and changes in PSA, and may be helpful for treating men with localized PC. Based on our data, we suggest that conservation management might be suitable for patients at low or intermediate risk associated with tumor involvement ≤5% in TURP specimens, PSA_TURP ≤ 4 ng/mL, and ≥68% PSA reduction following TURP. Moreover, for patients with no pre-TURP PSA, GS < 7 and low PSA_TURP could potentially be utilized to select which patients could be considered for conservative management after TURP. Therefore, tumor involvement in TURP specimens and changes in PSA and PSA_TURP could be used as adjuvant markers to guide the implementation of a risk-adaptive strategy for selected PC patients.

## Figures and Tables

**Table 1:** Adjusted hazard ratio of determine factors associated with the biochemical progression-free survival for 687 patients.

Variable	HR	95% CI	*p* Value
Age			
<75	Ref		
≥75	1.01	0.75–1.35	0.98
Gleason score (GS)			
<7	Ref		
≥7	1.78	1.27–2.50	0.001 *
Clinical stage			
T1	Ref		
T2	0.98	0.73–1.32	0.91
PSA at diagnosis			
≤10	Ref		
>10	2.18	1.66–3.41	<0.001 *
Cancer in TURP			
≤5%	Ref		
>5%	2.24	1.62–3.11	<0.001 *
Treatment			
Local therapy	Ref		
Conservative Tx	3.34	2.41–4.63	<0.001 *

* Statistical significance.

**Table 2:** Characteristics of 362 T1-2N0M0 patients who underwent TURP at diagnosis and recheck PSA after TURP at diagnosis.

	No. of Patients		
	PSA_TURP ≤ 4	PSA_TURP > 4	*p* Value
Patients	228	134	
Age			0.064
<75 y/o	120	57	
≥75 y/o	108	77	
PVR (≥100 mL)			0.007
No	158	110	
Yes	70	24	
GS			0.039
<7	126	59	
≥7	102	75	
Cancer in TURP			0.077
≤5%	124	57	
>5%	93	64	
Unknown	11	13	
PSA at Diagnosis			<0.001
≤10	128	40	
>10	100	94	
Treatment			<0.001
Local therapy	79	76	
Conservative Tx	149	58	
Biochemical failure			<0.001
No	176	70	
Yes	52	64	
Disease failure ^a^			0.013
No	201	105	
Yes	27	29	
Survival status			0.005
Dead	45	44	
Alive	183	90	
Prostate cancer-specific survival			0.004
Dead	8	15	
Alive	220	119	

^a^ = loco-regional recurrence and/or distant metastasis.

**Table 3:** Characteristics of patients with stage T1-2N0M0 who underwent TURP at diagnosis.

Characteristics	Number
Age	
Range	45~88 (years)
Median	73.12 (years)
<75	463 (55%)
≥75	383 (45%)
Gleason score	
<7	355 (42%)
≥7	477 (56%)
Unknown	14 (1.7%)
Clinical T stage	
T1	441 (52%)
T2	405 (48%)
Cancer in TURP specimen	
Cancer (−)	159 (19%)
Cancer (+)	687 (81%)
PSA at diagnosis	
≤10	338 (40%)
>10	508 (60%)
Treatment	
Local therapy	359 (42%)
Conservative Tx	487 (58%)

## Data Availability

Data availability is limited due to institutional data protection law and confidentiality of patient data.

## Supplementary Materials

The following are available online at https://www.mdpi.com/2072-6694/13/1/74/s1, Table S1: Characteristics of 687 T1–2N0M0 patients who underwent TURP at diagnosis, Figure S1: Kaplan-Meier PFS survival curves of 687 patients stratified by risk groups.
