# Supplementary material for: The Predictive Role of Prostate-Specific Antigen Changes Following Transurethral Resection of the Prostate for Patients with Localized Prostate Cancer
Source: Cancers (Basel). 2020 Dec 29;13(1):74. doi: 10.3390/cancers13010074 (PMC7796215; doi:10.3390/cancers13010074)

Table S1 Characteristics of 687 T1-2N0M0 patients who underwent TURP at diagnosis

	No. of patients		
	Local therapy	Conservative management	*p* value
**Patients**	327 (100%)	360 (100%)	
**Age**			<0.001
<75y/o	220 (67%)	172 (48%)	
≥ 75y/o	107 (33%)	188 (52%)	
**GS**			<0.001
<7	98 (30%)	190 (53%)	
≥7	229 (70%)	170 (47%)	
**PSA at Diagnosis**			<0.001
≤10	93 (28%)	162 (45%)	
>10	234 (72%)	198 (55%)	
**Clinical stage**			<0.001
T1	131 (40%)	213 (59%)	
T2	196 (60%)	147 (41%)	
**Biochemical failure**			<0.001
No	259 (79%)	202 (56%)	
Yes	68 (21%)	158 (44%)	
**Disease failure**[a]			0.004
No	273 (83%)	268 (74%)	
Yes	54 (17%)	92 (26%)	
**Survival status**			<0.001
Dead	67 (20%)	145 (40%)	
Alive	260 (80%)	215 (60%)	
**Prostate cancer-specific survival**			<0.001
Dead	16 (5%)	45 (13%)	
Alive	311 (95%)	315 (87%)	

* Statistical significance

[a]= loco-regional recurrence and/or distant metastasis

Figure S1 Kaplan-Meier PFS survival curves of 687 patients stratified by risk groups

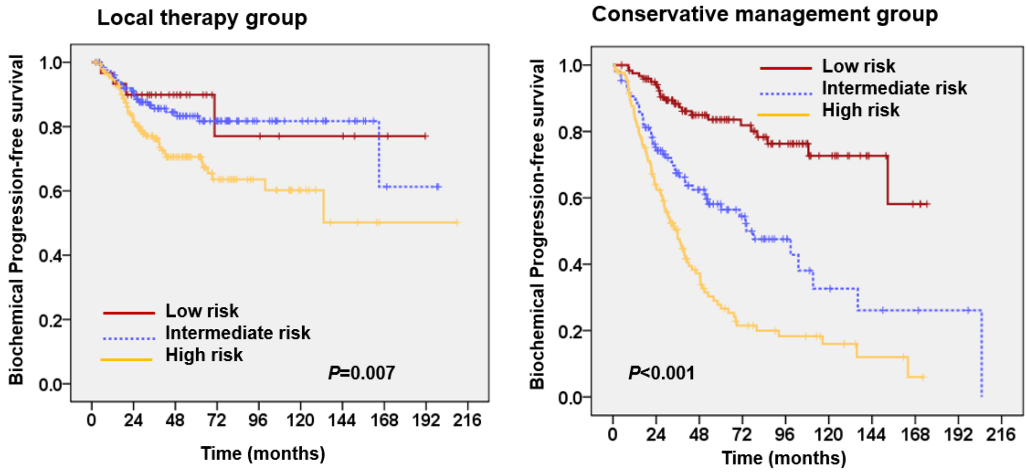

Supplement: Supplementary file 1 [file cancers-13-00074-s001.pdf]
